# Associations between hepatic miRNA expression, liver triacylglycerols and gut microbiota during metabolic adaptation to high-fat diet in mice

**DOI:** 10.1007/s00125-017-4209-3

**Published:** 2017-01-19

**Authors:** Vincent Blasco-Baque, Berengère Coupé, Aurelie Fabre, Sandra Handgraaf, Pierre Gourdy, Jean-François Arnal, Michael Courtney, Carole Schuster-Klein, Beatrice Guardiola, François Tercé, Rémy Burcelin, Matteo Serino

**Affiliations:** 1 Institut National de la Santé et de la Recherche Médicale (Inserm), Toulouse, France; 2 Unité Mixte de Recherche, Institut de Maladies Métaboliques et Cardiovasculaires (I2MC), Université Paul Sabatier (UPS), Toulouse, France; 3 Faculté de Chirurgie Dentaire de Toulouse, Université Paul Sabatier, Toulouse, France; 4 Vaiomer SAS, Labège, France; 5 Pôle d’Innovation Thérapeutique Métabolisme, Recherche de Découvertes, Institut de Recherches Servier, Suresnes, France; 6 Unité Mixte de Recherche (UMR) 1220, Institut de Recherche en Santé Digestive (IRSD), Université Paul Sabatier (UPS), Centre Hospitalier Universitaire (CHU) Purpan, Place du Docteur Baylac, CS 60039, 31024 Toulouse, Cedex 3 France

**Keywords:** Gut microbiota, High-fat diet, Liver triacylglycerol content, Metabolic adaptation, Metabolic diseases, miRNA

## Abstract

**Aims/hypothesis:**

Despite the current pandemic of metabolic diseases, our understanding of the diverse nature of the development of metabolic alterations in people who eat a high-fat diet (HFD) is still poor. We recently demonstrated a cardio-metabolic adaptation in mice fed an HFD, which was characterised by a specific gut and periodontal microbiota profile. Since the severity of hepatic disease is characterised by specific microRNA (miRNA) signatures and the gut microbiota is a key driver of both hepatic disease and miRNA expression, we analysed the expression of three hepatic miRNA and studied their correlation with hepatic triacylglycerol content and gut microbiota.

**Methods:**

Two cohorts of C57BL/6 4-week-old wild-type (WT) male mice (*n* = 62 and *n* = 96) were fed an HFD for 3 months to provide a model of metabolic adaptation. Additionally 8-week-old C57BL/6 mice, either WT or of different genotypes, with diverse gut microbiota (*ob/ob*, *Nod1*, *Cd14* knockout [*Cd14*KO] and *Nod2*) or without gut microbiota (axenic mice) were fed a normal chow diet. Following which, glycaemic index, body weight, blood glucose levels and hepatic triacylglycerol levels were measured. Gut (caecum) microbiota taxa were analysed by pyrosequencing. To analyse hepatic miRNA expression, real-time PCR was performed on total extracted miRNA samples. Data were analysed using two-way ANOVA followed by the Dunnett’s post hoc test, or by the unpaired Student’s *t* test. A cluster analysis and multivariate analyses were also performed.

**Results:**

Our results demonstrated that the expression of miR-181a, miR-666 and miR-21 in primary murine hepatocytes is controlled by lipopolysaccharide in a dose-dependent manner. Of the gut microbiota, Firmicutes were positively correlated and Proteobacteria and *Bacteroides acidifaciens* were negatively correlated with liver triacylglycerol levels. Furthermore, the relative abundance of Firmicutes was negatively correlated with hepatic expression of miR-666 and miR-21. In contrast, the relative abundance of *B. acidifaciens* was positively correlated with miR-21.

**Conclusions/interpretation:**

We propose the involvement of hepatic miRNA, liver triacylglycerols and gut microbiota as a new triad that underlies the molecular mechanisms by which gut microbiota governs hepatic pathophysiology during metabolic adaptation to HFD.

**Electronic supplementary material:**

The online version of this article (doi:10.1007/s00125-017-4209-3) contains peer-reviewed but unedited supplementary material, which is available to authorised users.

## Introduction

The current pandemic of metabolic diseases, such as obesity and type 2 diabetes, cannot be completely explained by genetic alterations and the growing consumption of a Western diet [1, 2]. Moreover, obesity is not an inevitable consequence of a fat-rich diet, since both people and mice consuming a high-fat diet (HFD) can display the opposite metabolic outcome, suggesting the existence of metabolic adaptations in some individuals [3, 4]. Among the factors that may affect the metabolic processes on an individual basis [5] are the gut microbiota [6], the impact of which on host metabolism has been established [7–9]. We previously found that adaptation to obesity in terms of insulin sensitivity was characterised by a specific gut microbiota profile in insulin-resistant vs insulin-sensitive obese individuals [10]. In addition, we showed that divergent gut microbiota profiles characterise the different metabolic phenotypes developed during metabolic adaptation to an HFD in mice [3, 4]. Recently, we reported that the periodontal microbiota profile correlates with cardio-metabolic adaptations to an HFD in mice [11].

Furthermore, along with xenobiotics [12], diet is considered to be the strongest modulator of gut microbiota [13]. Evidence from hepatic transcript profiles in mice has suggested that liver pathophysiology may be affected during metabolic adaptation to HFD in mice [14]. The existence of a gut–liver axis has been previously demonstrated and the liver is the organ in which xenobiotic metabolism occurs, especially with regard to our capacity of responding to gut microbial antigens [15].

Moreover, the alteration of gut microbiota, termed dysbiosis, is an additional causal factor in the development of hepatic steatosis [16], a condition that involves the accumulation of hepatic triacylglycerols, which is a common feature of metabolic disease [17]. Indeed, the different stages of hepatic diseases, including steatosis, hepatitis and hepatocellular carcinoma (HCC), are identifiable by a precise microRNA (miRNA) signature [18]. miRNA are pleiotropic modulators of gene expression [19] that have been shown to be under the control of gut microbiota [20]. Some miRNA, for example miR-181a, miR-666 and miR-21, are specifically involved in the modulation of liver pathophysiology [18, 21].

In the present study, we aimed to elucidate the gut microbiota profiles that are associated with metabolic adaptations to HFD in mice. We also aimed to investigate the associations between specific taxa of gut microbiota and hepatic expression of miR-181a, miR-666 and miR-21 in mouse models of hepatic steatosis. In addition, we explored the link between miRNA expression levels and metabolic parameters, such as glucose tolerance, body weight and fasting blood glucose.

## Methods

### Animal models and dietary treatment

All animal experimental procedures were approved by the local ethical committee of Rangueil University Hospital (Toulouse, France). All experimenters were blind to group assignment and outcome assessment. No data, samples or animals were excluded from this study.

#### Animal model for metabolic adaptation to HFD

An initial cohort of 62 and a second cohort of 96 C57BL/6 4-week-old wild-type (WT) male mice (Charles River, L'Arbresle, France) were fed an HFD (∼72% fat [corn oil and lard], 28% protein and <1% carbohydrate; SAFE, Augy, France) for 3 months [4]. Mice were housed in groups (10–11 mice per cage) in a specific pathogen-free controlled environment (inverted 12 h light cycle; lights off at 10:00 hours). Mice were killed by cervical dislocation after a 6 h fast. Tissues were collected and snap frozen in liquid nitrogen.

#### Axenic, WT, *ob/ob*, *Nod1*, *Cd14*KO and *Nod2* mice

Eight-week-old C57BL/6 mice (Charles River) either WT or of different genotypes, with diverse gut microbiota (*ob/ob*, *Nod1*, *Cd14* knockout [*Cd14*KO] and *Nod2*) or without gut microbiota (axenic mice) were fed a normal chow diet. Mice were housed in groups (4–6 mice per cage) in a specific pathogen-free controlled environment (inverted 12 h light cycle; lights off at 10:00 hours). After the mice were killed, the livers were collected and snap frozen in liquid nitrogen and stored at −80°C until analysis.

### GTT and hepatic triacylglycerol measurement

After 3 months of HFD, an IPGTT or OGTT were performed. Briefly, for the IPGTT, 6 h fasted mice were injected with glucose (1 g/kg) into the peritoneal cavity, as previously described [22]. An OGTT was performed via oral administration of glucose (1.5 mg/g) following a 6 h fast. Blood glucose levels were measured 30 min before glucose administration and at 0, 15, 30, 60, 90 and 120 min following glucose challenge.

For both IPGTT and OGTT, the glycaemic index was calculated as the sum of the blood glucose values (mmol/l) divided by the total time of the curve in min to present value in mmol/l × min, or additionally multiplied by 1000 to give value in μmol/l × min.

Liver triacylglycerol content was measured by a colorimetric assay using free glycerol and triacylglycerol reagents (Sigma Aldrich, St Louis, MO, USA) and the plate was read using the Multiskan Spectrum plate reader and the SkanIt RE software (both Thermo Labsystems, Beverly, MA, USA).

### Taxonomic analysis of gut microbiota by pyrosequencing

Caecum total DNA was extracted as previously described [4]. The whole 16S bacterial ribosomal RNA V2 region was targeted by the 28F-519R primers (designed by Research and Testing Laboratory [www.researchandtesting.com/, accessed 1 September 2016; Lubbock, TX, USA]) and pyrosequenced by the 454 GS FLX+ system (Roche, Branford, CT, USA) at the Research and Testing Laboratory. On average, 3000 sequences were generated per mouse. The minimum number of sequences guaranteed per mouse was 1606 (*n* = 62).

### Preparation of murine primary hepatocytes and lipopolysaccharide stimulation

Hepatocytes were isolated by a non-recirculating collagenase perfusion through the portal vein of anesthetised 8-week-old C57BL/6 WT or *Cd14*KO male mice fed a normal chow diet. Isolated cells were filtered through a 100 μm pore mesh nylon filter and cultured (2.5 × 10^6^ cells per well) onto 96-well plates in DMEM (BE12-614F; Lonza, Levallois, France) supplemented with 10% (vol./vol.) FCS, 1% (vol./vol.) penicillin/streptomycin and 0.2 nmol/l l-glutamine. After 12 h, the medium was replaced with medium plus industrially purified lipopolysaccharide (LPS; Sigma Aldrich, St Louis, MO, USA) either from the proinflammatory *Escherichia coli* serotype O55:B5 [22], or the *E. coli* strain O111:B4, which stimulates human hepatocytes [23]. Two doses of LPS were tested: 10 ng/ml (low dose) and 100 ng/ml (high dose), and cells were stimulated for 6 h. Experiments were performed in quadruplicates (control) or pooled duplicates (LPS).

### miRNA-based quantitative PCR

Real-time PCR for miRNA expression was performed on total miRNA extracted from cells or livers using the miRNeasy kit (Qiagen, Courtaboeuf, France). The expression of each miRNA was normalised to U6 small nuclear RNA (snRNA) expression [23]. For in vitro analyses, cells were directly harvested into Qiazol solution, which was provided with the miRNeasy kit. For ex vivo analyses, frozen pieces of liver were put directly into the Qiazol and total miRNA was extracted following the manufacturer’s protocol. Expression values were quantified using the ${2}^{-\Delta \Delta {\mathrm{C}}_{\mathrm{t}}}$ method [4].

### Hepatic microarray analysis

Total RNA was isolated from the right lobe of the liver using TRIzol (Life Technologies, Villebon sur Yvette, France) according to the manufacturer’s protocol. Preparation, labelling and hybridisations of cDNA were performed as per the manufacturer’s protocol. Samples were analysed using an Agilent SurePrint G3 Mouse GE 8 × 60K chip (design 028005; Agilent Technologies, Courtaboeuf, France). The hybridised microarrays were washed and scanned using an Agilent G2505C scanner. Data were extracted from the scanned image using the Agilent Feature Extraction software version 10.10.1.1. All of these steps were performed at the GENOTOUL GeT-TRIX facility at the French National Institute for Agricultural Research (INRA; Toulouse, France).

### Statistical analysis

Statistical analyses were performed by two-way ANOVA followed by the Dunnett’s post hoc test, or using the unpaired Student’s *t* test, using GraphPad Prism version 7.00 for Windows 7 (GraphPad, San Diego, CA, USA). A *p* value <0.05 was considered significant. Cluster analysis was performed using PermutMatrixEN software (http://download.cnet.com/PermutMatrix/3000-20432_4-75325452.html, accessed 20 June 2016) [24]. Multivariate analyses were performed using the Spearman correlation coefficient and *p* values were adjusted using the Benjamini–Hochberg correction (available at www.marum.de/Binaries/Binary745/BenjaminiHochberg.xlsx, accessed 12 November 2016). A String analysis was performed to study the network of genes targeted by miR-21; these genes were identified using the software miRTarBase (http://mirtarbase.mbc.nctu.edu.tw/, accessed 20 June 2016) [25].

## Results

### Hepatic triacylglycerol content correlates with metabolic adaptation to HFD feeding in mice

**Fig. 1:**
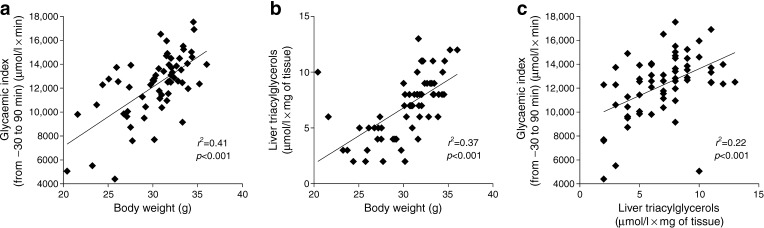
Metabolic diversity in C57BL/6 4-week-old WT male mice (*n* = 62) fed an HFD for 3 months. Three main metabolic variables are shown: glycaemic index (−30 to 90 min) during an IPGTT [4], body weight and liver triacylglycerols. Correlations between (**a**) glycaemic index vs body weight, (**b**) liver triacylglycerols vs body weight and (**c**) glycaemic index vs liver triacylglycerols

We aimed to investigate the effect of diabetogenic/non-obesogenic HFD in hepatic steatosis. To do this, hepatic triacylglycerol content was analysed in a murine model of metabolic adaptation to HFD [4, 11]. Glycaemic index (evaluated using IPGTT; Fig. 1a,c), body weight (Fig. 1a,b; trend over 3 months reported in ESM Fig. 1) and liver triacylglycerol content (Fig. 1b,c) were significantly correlated with each other. Liver triacylglycerol content showed high interindividual variation, which is typical of this animal model and suggests that liver triacylglycerol levels are dependent on the metabolic response of each mouse to HFD feeding (Fig. 1). Thus, we further confirm our previous findings from an analysis of hepatic gene expression during metabolic adaptation to HFD in mice, reinforcing the reproducibility of the animal model used in this study [14].

### Specific bacterial groups in the gut microbiota are associated with hepatic triacylglycerol content during metabolic adaptation to HFD feeding in mice

**Fig. 2:**
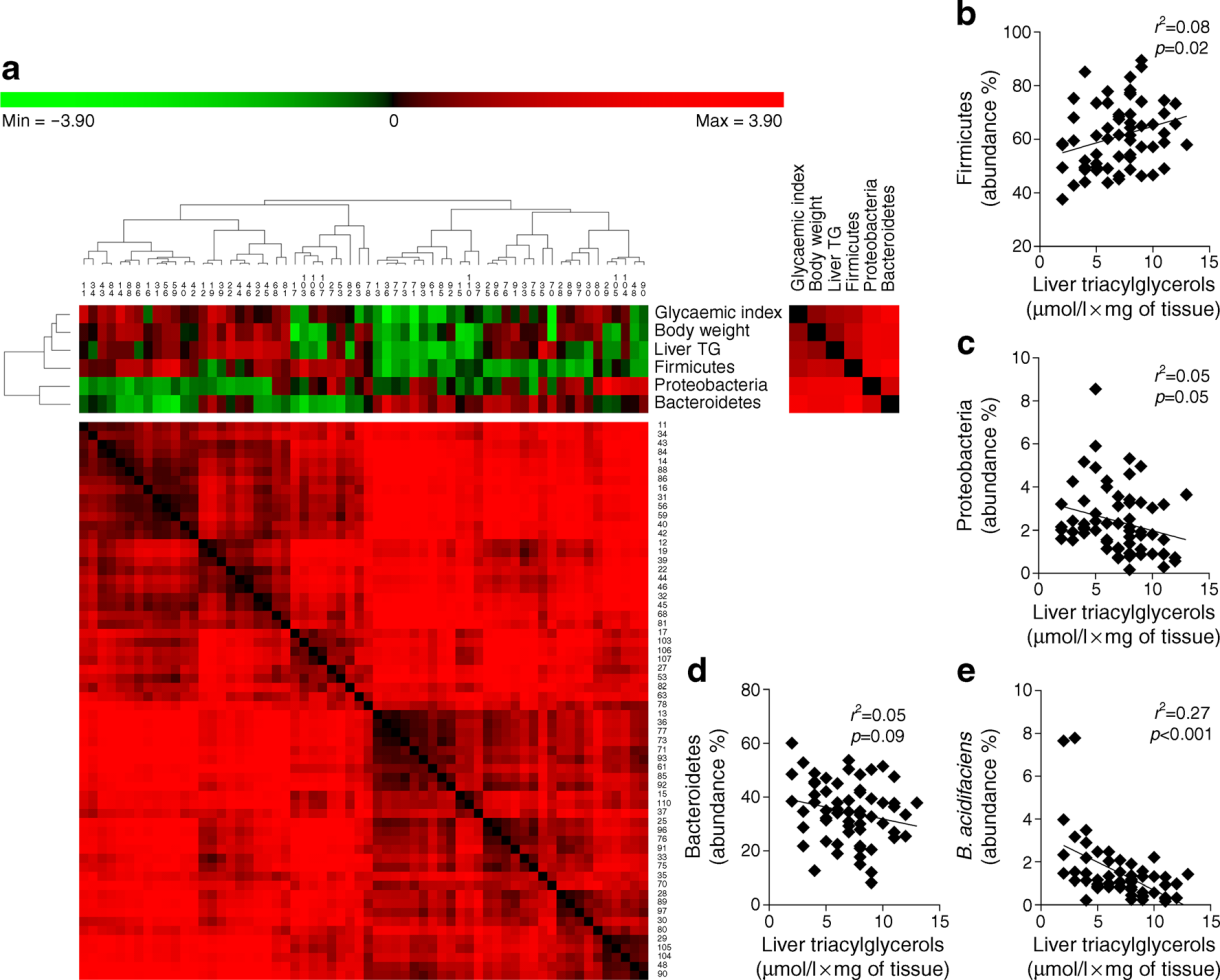
Integration data analysis between metabolic and metagenomic variables during metabolic adaptation to an HFD. (**a**) Heat map between metagenomics data (phylum level) and metabolic variables (glycaemic index, body weight and liver triacylglycerols [TG]). The dendrogram on the left-hand side of the heat map identifies clusters of metabolic and metagenomic variables; the dendrogram above the heat map identifies clusters of mice (the identification numbers are presented below this dendrogram). Cluster analysis was performed using PermutMatrixEN software [24]. Correlation matrices for the overall variables (right-hand side of the heat map) and mice (below the heat map) are also shown. Correlation analyses between liver triacylglycerols and the relative abundance (%) of (**b**) Firmicutes, (**c**) Proteobacteria, (**d**) Bacteroidetes and (**e**) *B. acidifaciens. n* = 62

We previously demonstrated that a specific gut microbiota profile characterises the mouse model of metabolic adaptation to HFD used in this study [4]. Hence, since the gut–liver axis manages our capacity to sense gut microbes [15], we investigated whether bacterial groups from the gut microbiota may be associated with the diversity of hepatic triacylglycerol content in HFD-fed mice. We sequenced the gut microbiota from all mice and performed a non a priori-based analysis to identify putative correlations between bacterial phyla and metabolic variables of interest. Out of the total 12 phyla identified, three were present in all mice: Firmicutes, Proteobacteria and Bacteroidetes (Table 1). As presented in Fig. 2a, all of the metabolic variables of interest (glycaemic index, body weight and liver triacylglycerol) were positively associated with Firmicutes, and negatively associated with Proteobacteria and Bacteroidetes. We challenged these associations by performing single linear regression analyses and found that Firmicutes showed a significant positive correlation with liver triacylglycerol content (Fig. 2b) and Proteobacteria showed a significant negative correlation with liver triacylglycerols (Fig. 2c). In contrast, single linear regression analysis revealed that Bacteroidetes showed a non-significant correlation with liver triacylglycerol content (Fig. 2d).

**Table 1:** Correlations of bacterial phyla/species with liver triacylglycerol content

Taxonomic category	Spearman correlation	
	Adjusted *p* value	*r* ^*2*^
Phyla		
Firmicutes	0.03^†^	0.09
Proteobacteria	0.02^†^	0.10
Bacteroidetes	0.05	0.05
Species		
*Bacteroides acidifaciens*	0.0021^†^	0.25
*Clostridium lactatifermentans*	0.0042^†^	0.14
*Tannerella* spp.	0.0063^†^	0.12
*Clostridium indolis*	0.0083	0.10
*Oscillibacter* spp.	0.0104	0.08
*Clostridium orbiscindens*	0.0125	0.08
*Alistipes* spp.	0.0146	0.08
*Ruminococcus* spp.	0.0167	0.07
*Clostridium aminophilum*	0.0188	0.06
*Eubacterium hallii*	0.0208	0.06
*Alistipes putredinis*	0.0229	0.06
*Eubacterium* spp.	0.0250	0.05
*Roseburia* spp.	0.0271	0.04
‘*Candidatus* Prevotella conceptionensis’	0.0292	0.04
*Oscillospira* spp.	0.0313	0.04
*Clostridium* spp.	0.0333	0.03
*Bacteroides* spp.	0.0354	0.03
*Alistipes shahii*	0.0375	0.03
*Odoribacter splanchnicus*	0.0396	0.02
*Parasutterella excrementihominis*	0.0417	0.02
*Alistipes finegoldii*	0.0438	0.002
*Oscillibacter valericigenes*	0.0458	0.02
*Butyrivibrio fibrisolvens*	0.0479	0.01
*Clostridium phytofermentans*	0.0500	0.001

*n* = 62

Spearman correlation adjusted according to the Benjamini–Hochberg correction for multiple comparisons (false-discovery rate <0.05)

^†^indicates *p* value is significant following Benjamini–Hochberg correction

We also tested whether specific bacterial species were associated with liver triacylglycerol levels. Of the 421 bacterial species identified in the gut microbiota, 24 were present in all mice (Table 1). Among those that were significantly correlated with liver triacylglycerol content, *Bacteroides acidifaciens* (belonging to the Bacteroidetes phylum) showed the most robust negative correlation (Fig. 2e).

In summary, these data identify that Firmicutes, Proteobacteria and *B. acidifaciens* of the gut microbiota correlate with hepatic triacylglycerol content during metabolic adaptation to HFD feeding in mice.

### Bacterial antigens drive hepatic miRNA expression in vitro

**Fig. 3:**
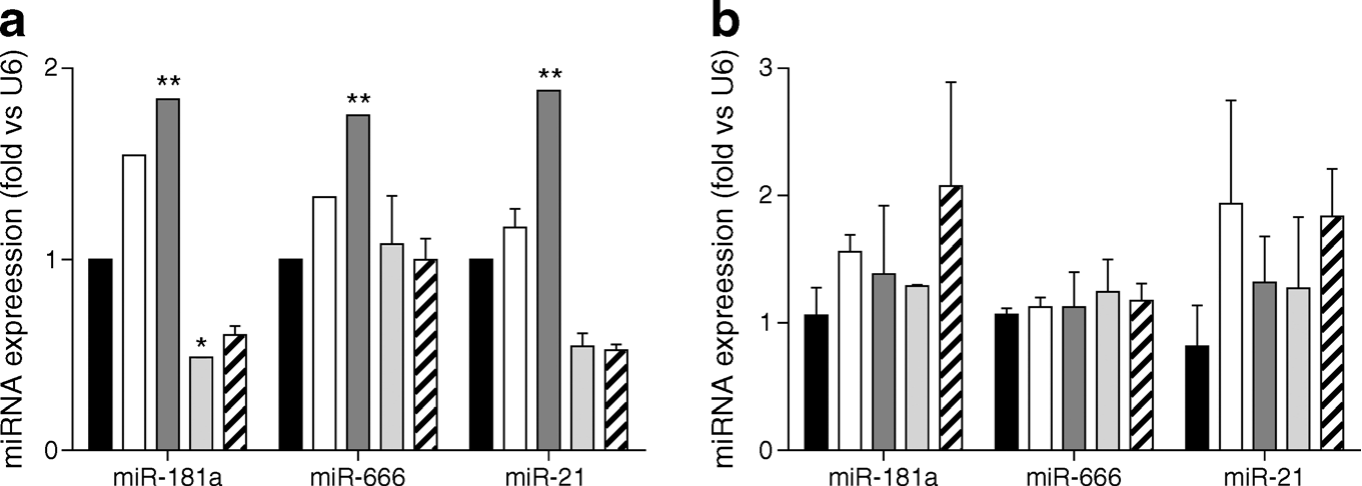
Real-time PCR miRNA analysis in 6 h LPS-stimulated primary murine hepatocytes from either WT or *Cd14*KO mice. miR-181a, miR-666 and miR-21 expression in primary murine hepatocytes from either (**a**) WT or (**b**) *Cd14*KO C57BL/6 male mice stimulated for 6 h with the purified LPS at various doses. The expression of each miRNA was normalised to U6 snRNA gene expression. Data are shown as mean ± SEM. Black bars, control (PBS [vehicle]); white bars, 10 ng/ml *E. coli* O55:B5 LPS ; dark grey bars, 100 ng/ml *E. coli* O55:B5 LPS; light grey bars, 10 ng/ml *E. coli* O111:B4 LPS; white bars with diagonal stripes, 100 ng/ml *E. coli* O111:B4 LPS. Two mice were used in two independent experiments to prepare primary hepatocytes; 2.5 × 10^6^ cells were plated per well; all experiments were performed in quadruplicates (control) and pooled duplicates (treatment). *p < 0.05, **p < 0.01 vs control; two-way ANOVA and Dunnett’s post hoc test

Gut microbiota dysbiosis can drive liver disease [26]; miRNA are known modulators of liver pathophysiology [18] and the gut microbiota modulates miRNA expression [20]. Therefore, we tested whether liver triacylglycerol content was associated with both gut microbiota and hepatic miRNA expression in a mouse model of metabolic adaptation to an HFD. First, we tested the capacity of bacterial antigens to modulate hepatic miRNA expression in vitro. The expression of three miRNA involved in the modulation of liver pathophysiology [18, 21], miR-181a, miR-666 and miR-21, was under the dose-dependent control of proinflammatory LPS from *E. coli* O55:B5 in primary WT murine hepatocytes. Also, 10 ng/ml LPS from the *E. coli* O111:B4 serotype, a known stimulator of nitric oxide synthase (NOS) in human hepatocytes [27], significantly decreased the expression of miR-181a, whilst treatment with 100 ng/ml showed a trend towards decreased expression of miR-181a and both 10 ng/ml and 100 ng/ml non-significantly decreased miR-21 expression. There was no significant effect of *E. coli* O111:B4 LPS on miR-666 levels (Fig. 3a). This LPS-induced modulation of miRNA expression was highly specific since it was not observed in primary hepatocytes from *Cd14*KO mice (Fig. 3b), known not to respond to LPS [22, 28].

**Fig. 4:**
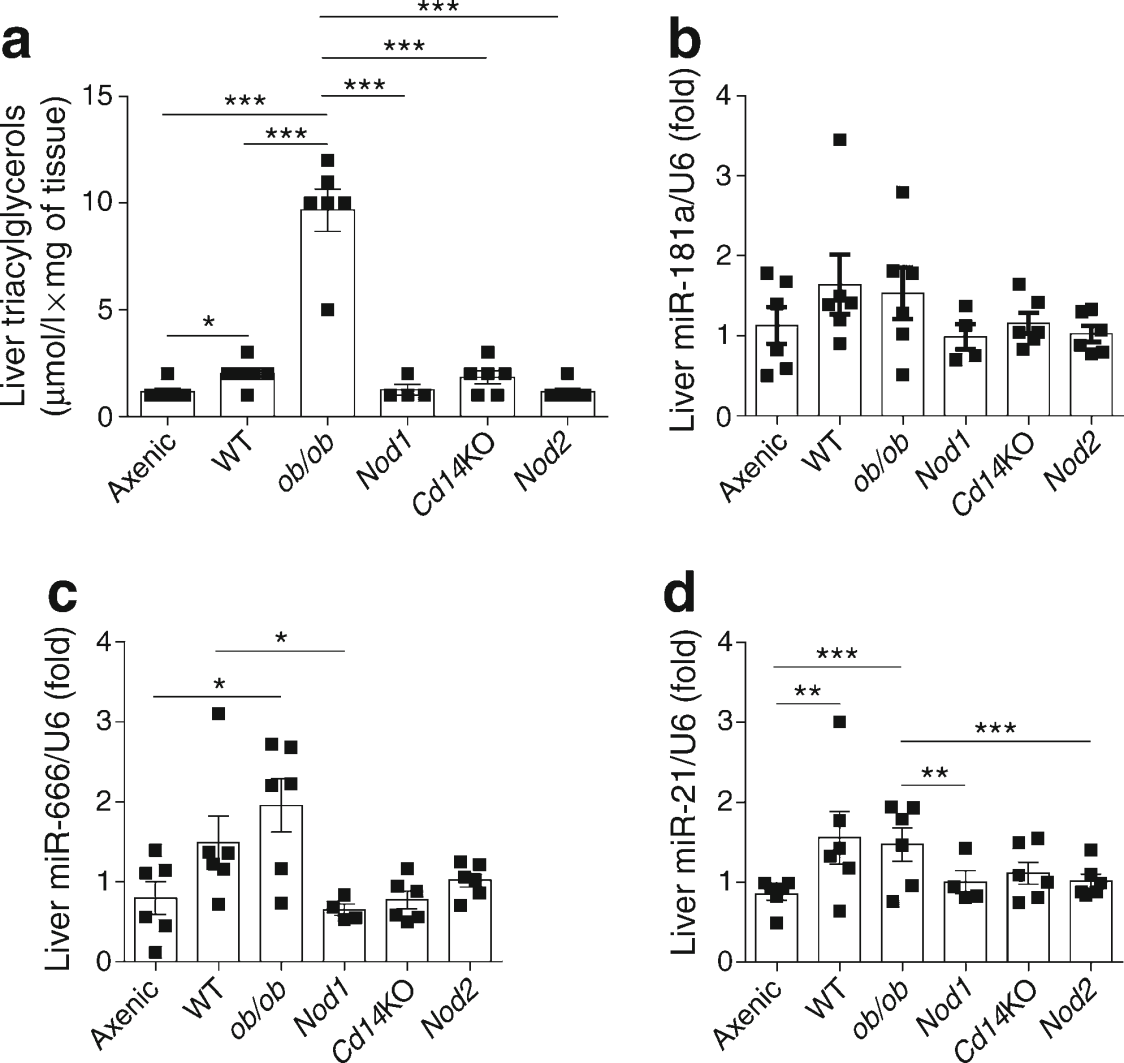
Analysis of triacylglycerol content and miRNA expression in the livers of 8-week-old C57BL/6 mice as models of gut microbiota dysbiosis: WT axenic, WT conventional (WT), *ob/ob*, *Nod1*, *Cd14*KO and *Nod2* mice fed normal chow. (**a**) Liver triacylglycerol content in each mouse model. Hepatic expression of (**b**) miR-181a, (**c**) miR-666 and (**d**) miR-21. The expression of each miRNA was normalised to U6 snRNA gene expression. Data are mean ± SEM; *p < 0.05, **p < 0.01, ***p < 0.001; Student *t* test

We then analysed the hepatic expression of the above miRNA in animal models of metabolic diseases characterised by gut microbiota dysbiosis, fed a normal chow diet (Fig. 4). First, we analysed the diversity of liver triacylglycerol content between the different models (Fig. 4a) and confirmed that the liver of axenic mice had lower triacylglycerol content than all other models [29]. With regard to miRNA expression, there was less variation in miR-181a expression between models suggesting this miRNA did not undergo metabolic adaptation and that there was no link with liver triacylglycerol levels (Fig. 4b). In contrast, the regulation of miR-666 and, to a larger extent, miR-21 expression was significantly altered depending on the animal model used (Fig. 4c,d).

Together these data show that: (1) in vitro, bacterial antigens control hepatic miRNA expression in a dose-dependent manner; and (2) in vivo, hepatic miRNA expression is associated with liver triacylglycerol content during gut microbiota dysbiosis.

### Hepatic miRNA expression is significantly associated with liver triacylglycerol content and with Firmicutes and *B. acidifaciens* relative abundance during metabolic adaptation to HFD feeding in mice

**Fig. 5:**
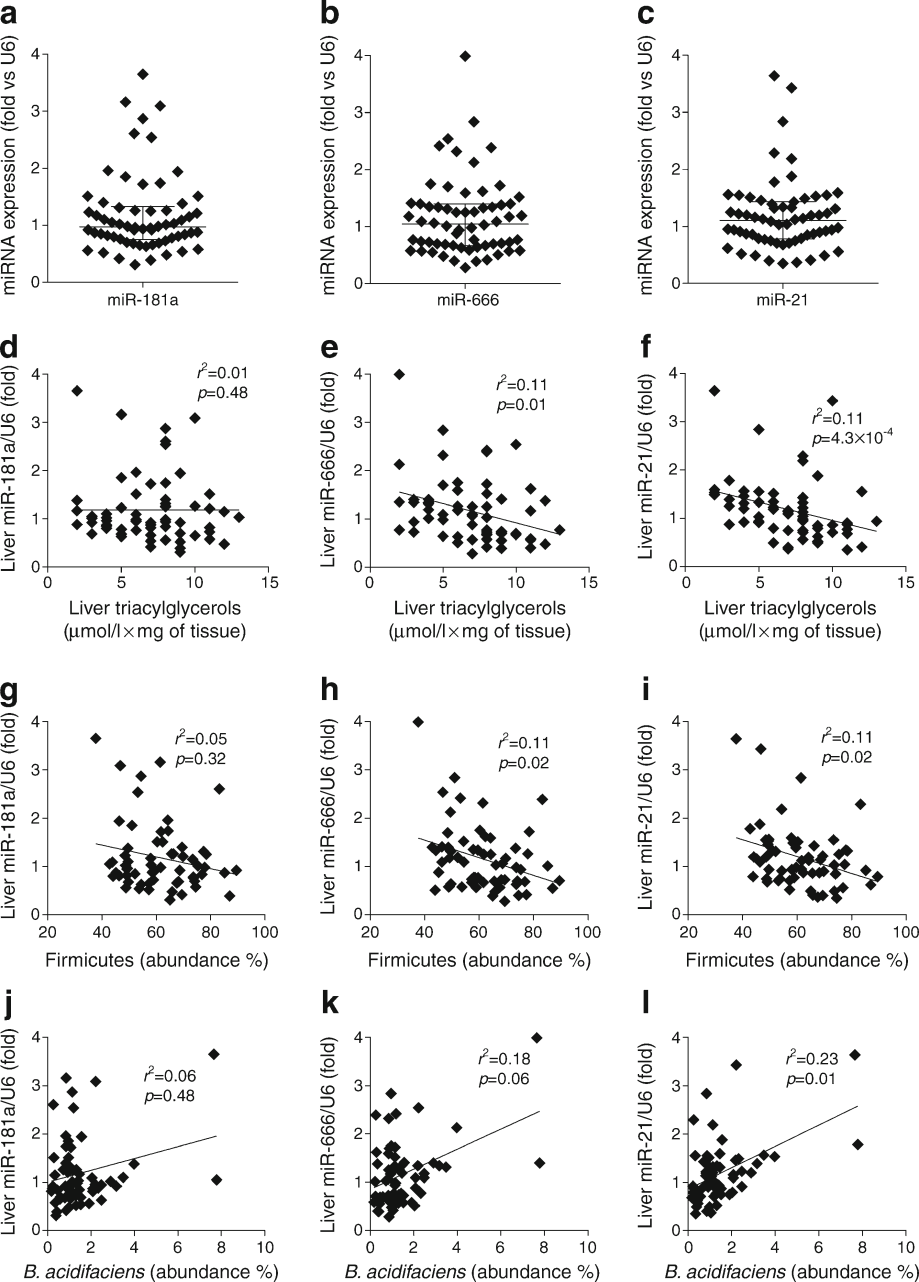
Hepatic miRNA expression analysis and correlation with liver triacylglycerols and gut microbiota during metabolic adaptation to HFD in mice. A cohort (*n* = 62) of C57BL/6 4-week-old WT male mice was fed an HFD for 3 months. Expression of (**a**) miR-181a, (**b**) miR-666 and (**c**) miR-21 was analysed. (**d–l**) Correlations between miRNA expression with (**d–f**) liver triacylglycerols, (**g–i**), Firmicutes relative abundance and (**j–l**) *B. acidifaciens* relative abundance. For (**e, f**), (**h, i**) and (**k, l**), significance determined by Spearman correlation adjusted according to the Benjamini–Hochberg correction for multiple comparisons (false-discovery rate <0.05)

The hepatic expression of miR-181a, miR-666 and miR-21 was studied in our model of metabolic adaptation to HFD. We observed a high diversity of expression for all three miRNA (Fig. 5a–c). Furthermore, miR-181a was not significantly correlated with liver triacylglycerols (Fig. 5d), whereas miR-666 and, to a larger extent, miR-21 showed significant correlations with this variable (Fig. 5e,f). Given that Firmicutes modulation is a marker of dysbiosis during metabolic adaptation (as we have already shown [4, 11]), we analysed whether this phylum may be correlated with hepatic miRNA expression in this model, along with *B. acidifaciens*. Of note, miR-181a was not significantly correlated with Firmicutes or *B. acidifaciens* relative abundance (Fig. 5g,j). In contrast, miR-666 was significantly correlated with Firmicutes but not with relative abundance of *B. acidifaciens* (Fig. 5h,k), whereas miR-21 was significantly correlated with both taxa (Fig. 5i,l).

In summary, hepatic miRNA expression is associated with both variations in liver triacylglycerol content and specific taxa of the gut microbiota during metabolic adaptation to HFD (Fig. 2 and Fig. 5).

### Hepatic miR-21-targeted genes and their metabolic relationships

**Fig. 6:**
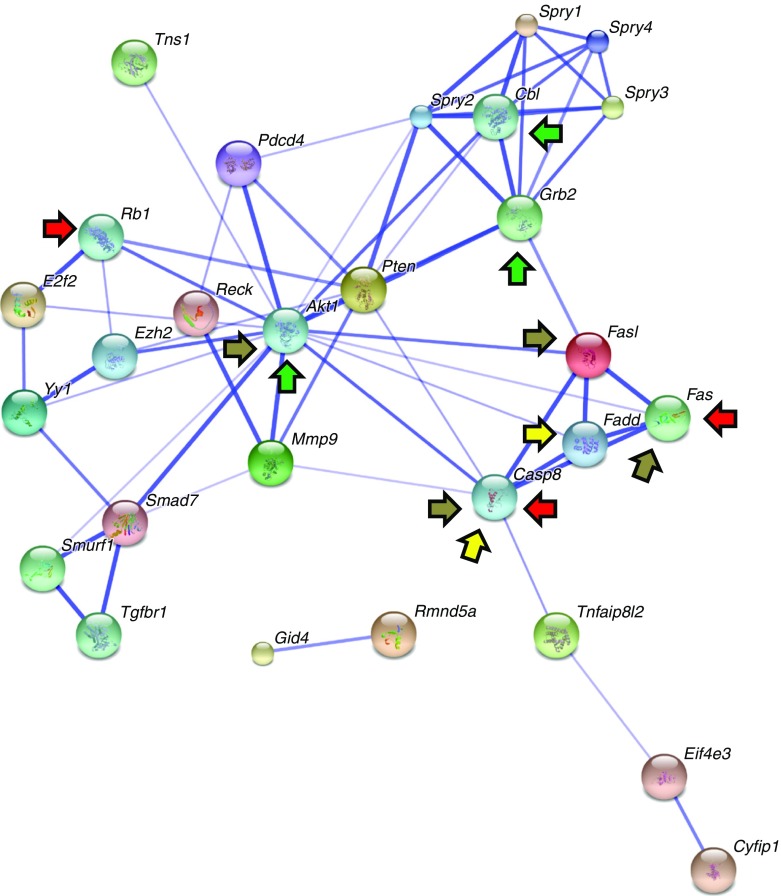
Network of genes targeted by miR-21, based on miRTarBase predictions and analysed by String. Arrows of different colours identify genes involved in specified pathways: red, hepatocyte apoptotic process; brown, NAFLD; green, insulin signalling; yellow, RIG-I-like receptor signalling pathway

To provide insight on the functional consequences of hepatic miR-21 expression on hepatic metabolic pathways, we performed a String analysis [30] of the network of genes targeted by this miRNA, which were identified by miRTarBase [25]. Notably, some genes targeted by miR-21 are involved in pathways associated with the hepatocyte apoptotic process, non-alcoholic fatty liver disease (NAFLD), insulin signalling and the proinflammatory RIG-I-like receptor signalling pathway (Fig. 6). This pathway regulates the production of proinflammatory cytokines, such as TNF-α and IL-8, in response to microbial antigens (i.e. LPS). Interestingly, we recently found that the RIG-I-like receptor signalling pathway is modulated in the periodontitis-induced periodontal dysbiosis during cardio-metabolic adaptation to HFD [11]. This result suggests an important association between the RIG-I-like receptor signalling microbial pathway and metabolic adaptation to an HFD in mice.

**Fig. 7:**
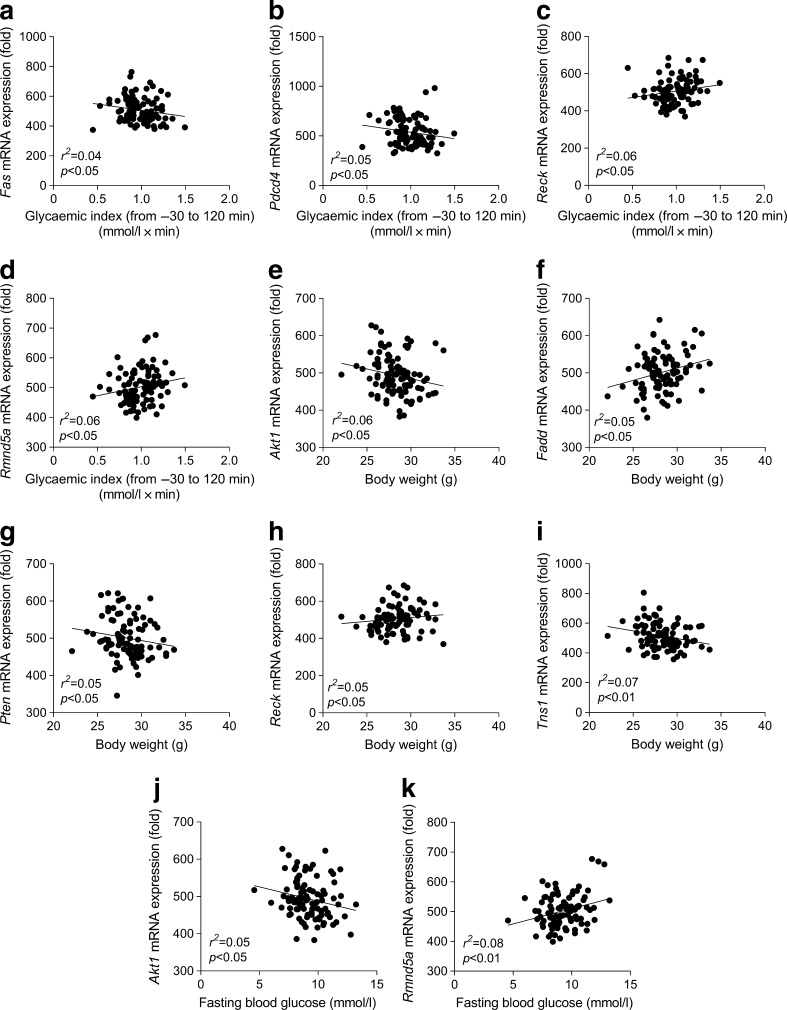
Correlations of genes targeted by miR-21 with metabolic variables during metabolic adaptation to a HFD in mice. (**a–k**) Correlation of specified genes with (**a–d**) glycaemic index (−30 to 120 min) during an OGTT, (**e–i**) body weight and (**j, k**) 6 h fasting blood glucose in an independent cohort of C57BL/6 4-week-old WT male mice (*n* = 96) fed a diabetogenic/non-obesogenic HFD for 3 months

Next, we investigated whether genes targeted by miR-21 may correlate with key metabolic variables: glucose tolerance, body weight and fasting blood glucose. To corroborate our observations in the first HFD-fed murine cohort, the correlation analysis was conducted in a second independent murine cohort (*n* = 96) of metabolic adaptation to HFD, in which we performed a liver microarray analysis. Importantly, the expression of *Fas*, *Pdcd4*, *Reck* and *Rmnd5a* was significantly correlated with the glycaemic index evaluated during an OGTT (Fig. 7a–d). Moreover, the expression of *Akt1*, *Fadd*, *Pten*, *Reck* and *Tns1* was significantly correlated with body weight (Fig. 7e–i). Additionally, the expression of *Akt1* and *Rmnd5a* was significantly correlated with fasting blood glucose (Fig. 7j,k).

Together these data suggest that the association of gut microbiota with hepatic miR-21 expression may have metabolic consequences via the modulation of genes targeted by miR-21.

## Discussion

In this study we report that, beyond the diversity already observed for blood glucose and body weight [3, 4, 11], liver triacylglycerol content is characterised by a high heterogeneity according to the individual response of mice to a diabetogenic/non-obesogenic HFD. Furthermore, liver triacylglycerol content was positively associated with the relative abundance of Firmicutes, and negatively associated with hepatic miR-21 expression and the relative abundance of Proteobacteria and *B. acidifaciens*.

Our finding that hepatic steatosis follows metabolic diversity on an individual basis, in mice fed a diabetogenic/non-obesogenic HFD, confirms our previous observations where hepatic lipid metabolism in this model, evaluated at the level of gene expression, was shown to be modulated according to the response to an HFD [14]. Therefore, our results reinforce the reproducibility of this animal model.

The observed positive association between hepatic triacylglycerol content and the relative abundance of Firmicutes is in contrast with the results of Henao-Mejia et al [26]. The authors found a reduction of Firmicutes in a model for inflammasome-mediated dysbiosis, regulating the progression of NAFLD and obesity [31]. However, the murine model used in our study is very different from the one used by Henao-Mejia and colleagues; we used C57BL/6 WT mice, whereas these authors used C56BL/6 *Nlrp6* KO mice. Therefore, it is likely that the different genetic backgrounds and genotypes of these mouse models account for disparity in the manifestations of dysbiosis, and are also responsible for the observed colitogenic phenotype. Differences in murine models may also explain the discrepancies found in the literature with regard to *B. acidifaciens.* This bacterium has recently been shown to be associated with liver disease [32], in contrast to our findings. Again, the murine model used in our study is very different from the mouse model used by Xie et al (streptozotocin/HFD-induced non-alcoholic steatohepatitis [NASH]/HCC C57BL/6 J mice) [32]. Thus, it is likely that under two very different dietary conditions, *B. acidifaciens* may have different associations with hepatic pathophysiology. This explanation is corroborated by another recent study by Yang et al that showed that *B. acidifaciens* prevents obesity and improves insulin sensitivity in mice [33]. Hence, based on the adaptation of both mice and microbes to a fatty environment, a divergent metabolic phenotype may arise, as we also recently reported with regard to cardio-metabolic adaptation to HFD in mice [11].

From a molecular perspective, miRNA represent promising molecules that may link gut microbiota dysbiosis to metabolic outcomes. Gut microbiota can modulate intestinal miRNA expression [20]. Moreover, a specific miRNA profile defines every stage of hepatic pathophysiology during the progression of disease from NAFLD (characterised by accumulated hepatic triacylglycerols) to NASH, (characterised by inflammation and fibrosis), up to HCC [18]. Thus, taking into account the gut–liver axis with regard to our capacity to sense gut microbes [15] and the fact that gut microbiota dysbiosis can drive NAFLD [25], it is plausible to consider that, in our study microbes, or even their antigens (e.g. LPS), may affect the liver via the modulation of hepatic miRNA expression, as we have shown here in vitro.

Specifically, miR-21 regulates both regeneration [34] and progression of fibrosis [35] in the liver, and long-term (18 weeks) inhibition of miR-21 reduces both body weight and adipocyte size in aged *db/db* mice [36]. These data are in accordance with the definition of miR-21 as a ‘disease miRNA’ [21]. However, this definition appears to be dependent on both animal model and diet. In fact, in the absence of HFD feeding, hepatic miR-21 expression is not correlated with hepatic triacylglycerols (Fig. 4; *r* ^*2*^ = 0.08, *p* = 0.1 [correlation not shown]). Notably, there is no clear evidence in the literature as to whether miR-21 may act as a marker of hepatic fat deposition. On one hand, Wu et al recently showed that miR-21 knockdown impairs lipid accumulation [37], which is in contrast to our findings. In contrast, Ahn et al reported that lycopene inhibits hepatic steatosis via the upregulation of miR-21, which is in accordance with our findings [38].

However, during metabolic adaptation to HFD, hepatic miR-21 expression was observed to be negatively associated with liver triacylglycerol content (Fig. 5f). This evidence suggests that during the adaptation to HFD, hepatic miRNA expression follows a different pattern of regulation. We confirmed this interpretation by showing that among the genes targeted by miR-21 are *Casp8* and *Fadd*, which are also part of the microbial RIG-I-like receptor signalling pathway. As previously mentioned, this pathway regulates the production of proinflammatory cytokines (such as TNF-α and IL-8) in response to microbial antigens (i.e. LPS). Importantly, we recently showed that the microbial RIG-I-like receptor signalling pathway is one of the most upregulated pathways during cardio-metabolic adaptation to HFD in mice [11], corroborating its implication in this phenomenon.

In conclusion, we propose a new triad linking gut microbiota, hepatic miRNA expression and liver triacylglycerol content. These findings may help to explain hepatic metabolism adaptation to an HFD in mice.

## Electronic supplementary material

Below is the link to the electronic supplementary material.

ESM Fig. 1 (PDF 35 kb)

## Abbreviations

*Cd14*KO: *Cd14* knockout
HCC: Hepatocellular carcinoma
HFD: High-fat diet
LPS: Lipopolysaccharide
miRNA: MicroRNA
NAFLD: Non-alcoholic fatty liver disease
NASH: Non-alcoholic steatohepatitis
snRNA: Small nuclear RNA
WT: Wild-type

## References

[1] Fretts AM, Follis JL, Nettleton JA (2015). Consumption of meat is associated with higher fasting glucose and insulin concentrations regardless of glucose and insulin genetic risk scores: a meta-analysis of 50,345 Caucasians. Am J Clin Nutr.

[2] Ruchat SM, Elks CE, Loos RJ (2009). Association between insulin secretion, insulin sensitivity and type 2 diabetes susceptibility variants identified in genome-wide association studies. Acta Diabetol.

[3] Burcelin R, Crivelli V, Dacosta A, Roy-Tirelli A, Thorens B (2002). Heterogeneous metabolic adaptation of C57BL/6J mice to high-fat diet. Am J Physiol Endocrinol Metab.

[4] Serino M, Luche E, Gres S (2012). Metabolic adaptation to a high-fat diet is associated with a change in the gut microbiota. Gut.

[5] Nicholson JK, Holmes E, Wilson ID (2005). Gut microorganisms, mammalian metabolism and personalized health care. Nat Rev Microbiol.

[6] Eckburg PB, Bik EM, Bernstein CN (2005). Diversity of the human intestinal microbial flora. Science.

[7] Backhed F, Ding H, Wang T (2004). The gut microbiota as an environmental factor that regulates fat storage. Proc Natl Acad Sci U S A.

[8] Ley RE, Backhed F, Turnbaugh P, Lozupone CA, Knight RD, Gordon JI (2005). Obesity alters gut microbial ecology. Proc Natl Acad Sci U S A.

[9] Turnbaugh PJ, Ley RE, Mahowald MA, Magrini V, Mardis ER, Gordon JI (2006). An obesity-associated gut microbiome with increased capacity for energy harvest. Nature.

[10] Serino M, Fernandez-Real JM, Garcia-Fuentes E (2013). The gut microbiota profile is associated with insulin action in humans. Acta Diabetol.

[11] Branchereau M, Reichardt F, Loubieres P (2016). Periodontal dysbiosis linked to periodontitis is associated with cardio-metabolic adaptation to high-fat diet in mice. Am J Physiol Gastrointest Liver Physiol.

[12] Maurice CF, Haiser HJ, Turnbaugh PJ (2013). Xenobiotics shape the physiology and gene expression of the active human gut microbiome. Cell.

[13] Carmody RN, Gerber GK, Luevano JM (2015). Diet dominates host genotype in shaping the murine gut microbiota. Cell Host Microbe.

[14] de Fourmestraux V, Neubauer H, Poussin C (2004). Transcript profiling suggests that differential metabolic adaptation of mice to a high fat diet is associated with changes in liver to muscle lipid fluxes. J Biol Chem.

[15] Szabo G, Bala S, Petrasek J, Gattu A (2010). Gut-liver axis and sensing microbes. Dig Dis.

[16] Dumas ME, Barton RH, Toye A (2006). Metabolic profiling reveals a contribution of gut microbiota to fatty liver phenotype in insulin-resistant mice. Proc Natl Acad Sci U S A.

[17] Forouzanfar MH, Alexander L, Collaborators GBDRF (2015). Global, regional, and national comparative risk assessment of 79 behavioural, environmental and occupational, and metabolic risks or clusters of risks in 188 countries, 1990-2013: a systematic analysis for the Global Burden of Disease Study 2013. Lancet.

[18] Wang XW, Heegaard NH, Orum H (2012). MicroRNAs in liver disease. Gastroenterology.

[19] Ambros V (2001). MicroRNAs: tiny regulators with great potential. Cell.

[20] Dalmasso G, Nguyen HT, Yan Y (2011). Microbiota modulate host gene expression via microRNAs. PLoS One.

[21] Androsavich JR, Chau BN, Bhat B, Linsley PS, Walter NG (2012). Disease-linked microRNA-21 exhibits drastically reduced mRNA binding and silencing activity in healthy mouse liver. RNA.

[22] Cani PD, Amar J, Iglesias MA (2007). Metabolic endotoxemia initiates obesity and insulin resistance. Diabetes.

[23] Boon RA, Iekushi K, Lechner S (2013). MicroRNA-34a regulates cardiac ageing and function. Nature.

[24] Caraux G, Pinloche S (2005). PermutMatrix: a graphical environment to arrange gene expression profiles in optimal linear order. Bioinformatics.

[25] Hsu SD, Lin FM, Wu WY (2011). miRTarBase: a database curates experimentally validated microRNA-target interactions. Nucleic Acids Res.

[26] Henao-Mejia J, Elinav E, Jin C (2012). Inflammasome-mediated dysbiosis regulates progression of NAFLD and obesity. Nature.

[27] Geller DA, Lowenstein CJ, Shapiro RA (1993). Molecular cloning and expression of inducible nitric oxide synthase from human hepatocytes. Proc Natl Acad Sci U S A.

[28] Luche E, Cousin B, Garidou L (2013). Metabolic endotoxemia directly increases the proliferation of adipocyte precursors at the onset of metabolic diseases through a CD14-dependent mechanism. Mol Metab.

[29] Velagapudi VR, Hezaveh R, Reigstad CS (2010). The gut microbiota modulates host energy and lipid metabolism in mice. J Lipid Res.

[30] Szklarczyk D, Franceschini A, Wyder S (2015). STRING v10: protein-protein interaction networks, integrated over the tree of life. Nucleic Acids Res.

[31] Elinav E, Strowig T, Kau AL (2011). NLRP6 inflammasome regulates colonic microbial ecology and risk for colitis. Cell.

[32] Xie G, Wang X, Liu P (2016). Distinctly altered gut microbiota in the progression of liver disease. Oncotarget.

[33] Yang JY, Lee YS, Kim Y (2016). Gut commensal Bacteroides acidifaciens prevents obesity and improves insulin sensitivity in mice. Mucosal Immunol.

[34] Song G, Sharma AD, Roll GR (2010). MicroRNAs control hepatocyte proliferation during liver regeneration. Hepatology.

[35] Zhang J, Jiao J, Cermelli S (2015). miR-21 inhibition reduces liver fibrosis and prevents tumor development by inducing apoptosis of CD24+ progenitor cells. Cancer Res.

[36] Seeger T, Fischer A, Muhly-Reinholz M, Zeiher AM, Dimmeler S (2014). Long-term inhibition of miR-21 leads to reduction of obesity in db/db mice. Obesity (Silver Spring).

[37] Wu H, Ng R, Chen X, Steer CJ, Song G (2016). MicroRNA-21 is a potential link between non-alcoholic fatty liver disease and hepatocellular carcinoma via modulation of the HBP1-p53-Srebp1c pathway. Gut.

[38] Ahn J, Lee H, Jung CH, Ha T (2012). Lycopene inhibits hepatic steatosis via microRNA-21-induced downregulation of fatty acid-binding protein 7 in mice fed a high-fat diet. Mol Nutr Food Res.

