# Supplementary material for: Associations between hepatic miRNA expression, liver triacylglycerols and gut microbiota during metabolic adaptation to high-fat diet in mice
Source: Diabetologia. 2017 Jan 19;60(4):690–700. doi: 10.1007/s00125-017-4209-3 (PMC6518927; doi:10.1007/s00125-017-4209-3)
Supplement: Supplementary file 1 — (PDF 35 kb) [file 125_2017_4209_MOESM1_ESM.pdf]

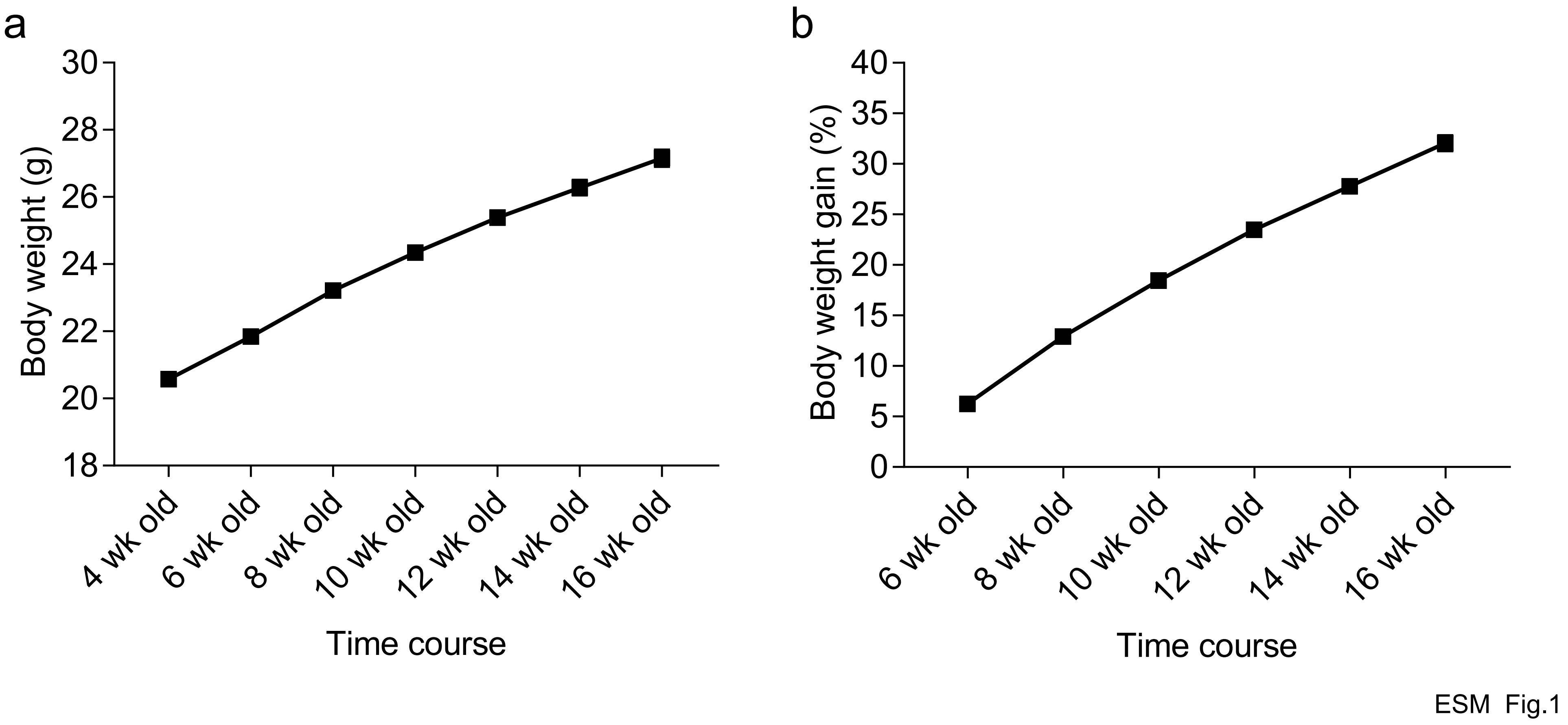

ESM Figure 1. Body weight trend over 3 months during metabolic adaptation to a HFD. Body weight trend in grams (a) and body weight gain as percentage (b).
